# Tomographic index as auxiliary criteria for surgery indication in fracture dislocation of acetabulum posterior wall

**DOI:** 10.1186/1755-7682-5-18

**Published:** 2012-06-20

**Authors:** Edison N Fujiki, Eduardo N Yamaguchi, Edison Miachiro, Takechi Chikude, Roberto Y Ikemoto, Luiz Carlos de Abreu, Vitor E Valenti, Luciano M R Rodrigues, Carlos B Monteiro, Carlo Milani

**Affiliations:** 1 Departamento de Cirurgia Ortopédica, Faculdade de Medicina do ABC, Santo André, SP, Brazil; 2 Laboratório de Escrita Científica, Departamento de Morfologia e Fisiologia, Faculdade de Medicina do ABC, Santo André, SP, Brazil; 3 Departamento de Fonoaudiologia, Faculdade de Filosofia e Ciências, Universidade Estadual Paulista, Marília, SP, Brazil; 4 Disciplina de Cirurgia Ortopédica, Faculdade de Medicina do ABC, Av. Príncipe de Gales, 821., 09060-650, Santo André, SP, Brazil

## Abstract

There are situations which the tomographic exam is done on the affected hip or situations where the contralateral hip presents abnormalities that make it impossible to compare. In this study we aimed to evaluate a tomographic index that does not require comparison between the both hips. Twenty two patients with unilateral acetabular fracture dislocation with fracture of posterior wall were studied. We established the relationship between the remaining posterior wall and the femoral head diameter (head/wall index-H/W index). We evaluated 45 two-dimensional computed tomography scan in normal hips and established the H/W index. In 45 normal hips we simulated a posterior wall fracture with involvement of 25% and 30% of the posterior wall and calculated the H/W index. We divided into five groups with five different H/W index (fractured group with non surgical treatment; fractured group; normal group; normal group with simulated fracture of 25% and; 30% of the posterior wall). 2.4 was the lowest limit of confidence interval of the group with 25% of the posterior wall fracture. When we analyzed the confidence interval of the 30% fracture group the upper limit of the confidence interval was 2.7, close to the lower limit of the surgical group that was 2.9. Thus, we suggest the 2.4 the H/W index limit as an auxiliary criteria to indicate whether or not to operate. H/W index is helpful to decide whether or not surgery indication in the fracture dislocation of the posterior wall of the acetabulum.

## Background

Bone fractures may lead to many impaired functions [1-3]. Acetabular posterior wall fracture is the most common type of acetabular fracture comprising around 18% to 33% of all cases [4-7]. It is becoming relatively frequent in orthopedists’ daily routine because traumatisms are increasing. Posterior wall fractures were classified by Judet-Letournel as one of the elementary types of acetabular fracture [4]. It appears to be simple on radiography; however, even after reduction it may present significant percentage of necrosis or it develops to hip arthrosis [6,8].

In our country, usually the first aid of these cases is done by orthopedists [7], who are often worried to solve emergency cases. Clinical examination to test the articular stability after reduction is the “gold” standard [9-15], however, in some cases these maneuvers are inconclusive or forgetfulness. Since the emergency was solved, the patient is evaluated by a hip surgeon specialist with the help of three standard plain radiographs (one anteroposterior and two Judet 45 degrees oblique pelvic radiographs) and a two-dimensional computed tomography (CT) scan. Nevertheless, if the fragment size is misestimate or it is difficult to evaluate even with a CT scan, and if there is no information regarding the hip stability criteria after reduction, certainly the decision to indicate or not indicate surgery will be difficult.

In the literature there are tomographic indexes that try to predict hip joint stability [9,16]; but all of these methods compare the fractured hip with the contralateral normal hip images. On the other hand, in medical practice, there are situations which the tomographic exam is done only on the affected hip, or even situations where the contralateral hip presents abnormalities that make it impossible to compare. Therefore, this investigation was undertaken to demonstrate the relationship between the tomographic index and instability.

## Methods

A retrospective study was done in 22 patients aged between 21 to 45 years old with unilateral posterior fracture dislocation of the acetabulum. The inclusion criteria were: joint instability, fractured fragment size, femoral head fracture, bone fragment interposition, residual subluxation and wall fracture impact displaced more than 2 mm. All patients were evaluated by a senior orthopedic hip surgeon, who analyzed the clinical history, three standard plain radiographs (one anteroposterior and two Judet 45 oblique pelvic radiographs), two-dimensional computed tomography scan and joint instability under anesthesia. All experimental protocols were approved by the ethics committee in research of our Institute.

After clinical examination and images evaluation it was decided whether or not to operate. Fourteen (63.64%) patients were operated and eight (36.36%) were not operated. Twelve of the 14 operated patients showed instability in the dynamic fluoroscopy examination under anesthesia (patient placed at supine position with neutral rotation and full extension of the hip; then the hip was flexed until 90 and adduction of 20 degrees). If the joint remained congruent in the anteroposterior and oblique in fluoroscopy projection it was considered stable. The pressure used on the maneuver was not considered due to the difficulty to evaluate it. The remaining two patients in spite of instability were operated because they presented large fractured size fragment on the posterior wall after image evaluation. The eight patients who were not operated presented stability in the examination under anesthesia and were considered by the senior surgeons as small size fractured fragment of posterior wall. Using CT (computed tomography) scan images we calculated the head wall index (H/W). The H/W index was calculated in 22 fractured hips and in 45 hips (26 patients were considered normal), we used 45 CT scans from hip images - from 26 patients - aged between 18 to 50 years old, which were considered normal (without fracture, osteoarthritis, dysplasia or other hip deformity). All images were obtained using a General Electric CT (two-dimensional computed tomography scan with 5 mm slice thickness).

In the control group we calculated the H/W index in the normal hip after we divided the posterior wall in percentages and the 25% and 30% of the distal posterior wall, respectively.

The tomographic images were digitized using a 7.2 mega pixels camera and transferred to the computer, studied and measured. We calculated the H/W index using the M2000 software for measuring angles and distances [17-20]. Two senior surgeons calculated the acetabular index for each image in all groups studied and average was calculated for each case.

**Figure 1:**
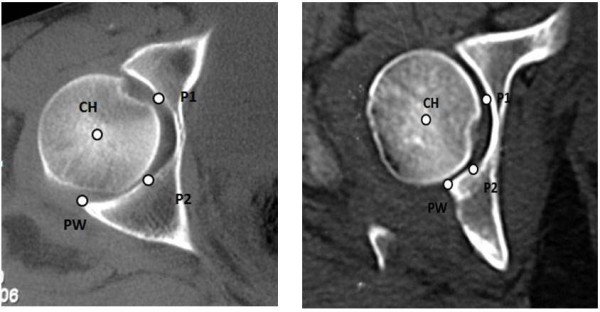
**In a non fractured acetabulum, four points were defined: P1→ anterior transition between the articular surface and the acetabular fossa.** P2 → posterior transition between the articular surface and the acetabular fossa. PW → posterior articular surface edge. CH →femoral head center.

From the axial CT slices through the acetabulum, in both normal and fractured hips, we had chosen the image that showed the largest anteroposterior femoral head diameter. We defined 4 points at this tomographic slice: point 1 (P1) corresponds to the anterior transition between the articular surface and the acetabular fossa; point 2 (P2) corresponds to the posterior transition between the articular surface and the acetabular fossa; the third point (PW) corresponds to the posterior articular surface edge, both in normal hips as in the remaining fractured wall; the fourth point (CH) corresponds to the femoral head center (Figure1).

**Figure 2:**
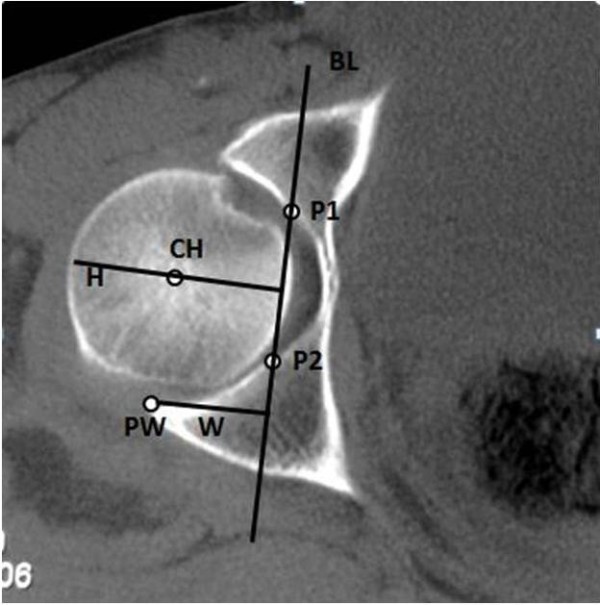
A straight base line (BL) was drawn throw the P1 and P2 points, and 2 other lines were drawn perpendicular to the BL line: Line W, from the PW point to BL line; and another line H, from the lateral edge of the femoral head, passing through CH point to BL line.

A straight baseline (BL) was drawn throw P1 and P2 points, and 2 other lines were drawn perpendicular to the BL: W line, from the PW to BL; and H line, from the lateral edge of the femoral head, passing through CH to BL (Figure2).

**Figure 3:**
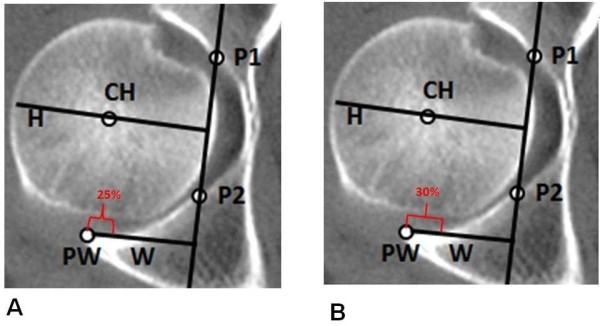
In normal CT scan group we divided the W line in percentages, we took the 25% (A) and 30% (B) of the distal wall and calculated the H/W index in remaining 75% and 70%, respectively.

The relationship between H and W lines (H/W index) was studied in normal hips and fractured cases. In fractured wall, the W line begins just in the fractured articular surface. After calculation of the H/W index in normal CT scan group, we divided the W line in percentages; we took the 25% distal of the wall and calculated the H/W index in the remaining 75% of the posterior wall, simulating a 25% (Figure3A) of the posterior wall fracture. The same procedure was done taking 30% (Figure3B) of distal W line and it was calculated the H/W index in the remaining 70%.

The tomographic acetabular index (H/W) of normal CT scan group was considered the *normal* group. The H/W index from the images after removing 25% and 30% of the posterior wall was named *minus 25%* (−*25%)* and *minus 30% (−30%*) groups, respectively. We called the group which underwent surgery the *surgical fracture* group while the group which the patients were not operated was named *conservative fracture group*.

**Figure 4:**
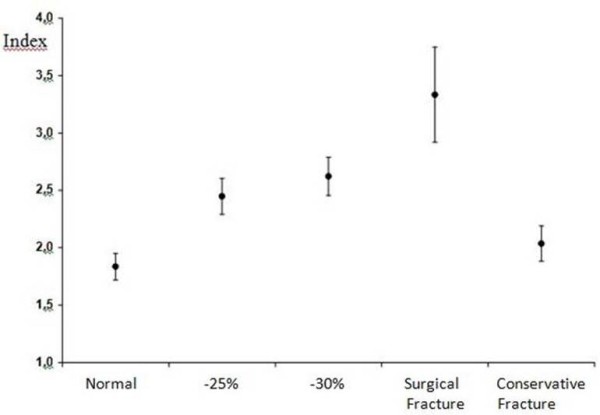
Means and standard deviations of the acetabular indexes observed in each group.

For all groups (fractured not operated group; fractured group, normal group, normal group with simulated fracture of 25% and 30% of the posterior wall) tomographic acetabular index and standard deviation were calculated (Table 1 and Figure4). The 95% confidence interval for each group was calculated (Table 2). The means of the indexes of all groups were compared by applying two-way ANOVA test followed by the post hoc Tukey multiple comparisons test. Differences were considered significant when the probability of a type I error was lower than 5% (p < 0.05).

**Table 1:** Means and standard deviations of the indexes observed of each group

Group	**Cases number**	**Acetabular index**	**Standard deviation**	**Minimum value**	**Maximum value**
Normal	45	1.8	0.116	1.7	2.1
−25%	45	2.4	0.156	2.3	2.8
−30%	45	2.6	0.166	2.4	3.0
Surgical fracture	14	3.2	0.440	2.9	3.9
Conservative fracture	8	2.1	0.130	1.9	2.2

**Table 2:** Confidence intervals (95%) of the mean acetabular index in each group

**Group**	**Confidence interval (95%)**	
	**Lower limit**	**Upper limit**
Normal	1.8	1.9
−25%	2.4	2.5
−30%	2.6	2.7
Surgical fracture	2.9	3.8
Conservative fracture	1.6	2.4

## Results

**Figure 5:**
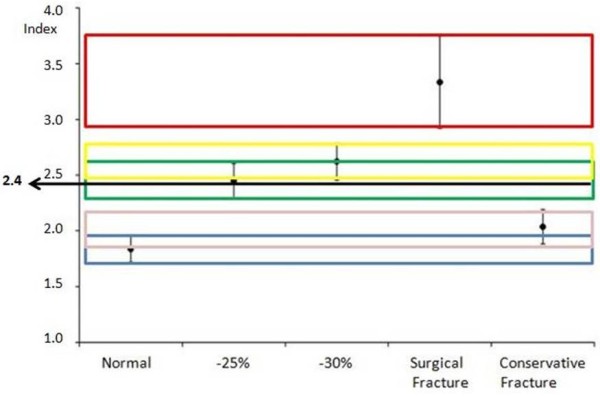
The relationship between the 2.4 index and the means and the standard deviation of the groups.

The H/W index presented a mean of 1.83 in the normal group while the conservative fracture group presented 2.08. The mean was 3.19 in the surgical fracture group and in the −25% and −30% groups the mean were 2.44 and 2.62, respectively. There were significant differences of mean H/W index among all groups, except in normal group vs. conservative fracture group (Table 3). When we analyzed the confidence interval of the H/W index we found that the upper limit of the conservative group was similar to the lower limit in the −25% group (H/W index: 2.4). The upper limit of the confidence interval of the −25% group was near the lower limit of the −30% group (H/W index: 2.49 and 2.57, respectively). Finally, the lower limit of the confidence interval of the surgical fracture group was close to the upper limit for the −30% group (Figure5).

**Table 3:** Descriptive levels of two way comparisons of the indices among five groups

**Comparison among the groups**		**Descriptive level (p-value)**
Normal x	−25%	< 0.001
Normal x	−30%	< 0.001
Normal x	Surgical fracture	< 0.001
Normal x	Conservative fracture	0.251
−25% x	−30%	< 0.001
−25% x	Surgical fracture	< 0.001
−25% x	Conservative fracture	<0.001
−30% x	Surgical fracture	< 0.001
−30% x	Conservative fracture	< 0.001
Surgical fracture x	Conservative fracture	< 0.001

## Discussion

Evaluations of hip instability in cases of fracture dislocation of the posterior wall are usually made by clinical examination after reduction under anesthesia, with hip flexion of 90° and slight adduction to test the stability in association to radiographic and tomographic analysis [16,21,22]. On the other hand, clinical evaluation sometimes is inconclusive, because in some cases it is associated with lesions, thereby losing this important parameter. Nonetheless, radiographic images in anteroposterior and oblique views may falsify the images of fragment sizes, depending on how they are situated in relation to the x-rays. Even tomographic images, which are more precise, may give rise to doubts regarding the sizes of fracture fragments, since the parameter of the size may be subjective, depending on each personal’s experience [16,21,22].

The method proposed by Calkins et al. [16] and Keith et al. [23] for tomographic measurements of instability classifies it into three groups: stable, indeterminate and unstable. Other study performed on cadavers [24] reported that osteotomy of the posterior wall lower than 25% did not affect the joint stability, while osteotomy higher than 50% of the posterior wall presented significant effect on joint stability. In our opinion the indeterminate group from 20 –25% to 40 –50% is the problem to make decision. Recently, Moed et al. [9] described a modified method, (alternative method) to calculate the instability and demonstrated that the new alternative method is more accurate than other methods used in the literature. However, all of these measurements may be impaired if the contralateral hip presents abnormalities such as anteversion, hip dysplasia, fractures or if the tomographic image was not digitized. Conversely, the H/W index takes into account the relative variation between the femoral head and the posterior wall in the affected hip, which is the real location of the problem and depends on the proportion head-wall where the instability was occurred. Moreover, the method proposed in the literature makes measurements comparing different hips and consequently the mistake rate may increase [21,22].

Olson et al. [21] reported that when the posterior wall was decreased by one third of size the remainder of the acetabulum was significantly overloaded when supported on one foot. In other words, even if the posterior wall loss did not cause joint instability it may be significant overloaded by the joint and it consequently cause early arthrosis. In our study there were two cases which the patients were operated without instability but the fragment was considered large by the senior surgeon.

We established a limit index of 2.4 for surgery or not surgery indication, stability or instability parameter, because the mean H/W index in the normal group and conservative group was similar. We did not observe statistical difference between these groups, however, when we analyzed the confidence interval, the upper limit of the conservative group was similar to the lower limit of the −25% group (2.4 H/W index) and there was statistical difference between conservative group and −25% group regarding the mean index. If surgery was not performed in subjects from 2.4 H/W index it was possible to mislead any case from this group. The end point of the upper limit of conservative group in our study was 2.4 H/W index, for this reason we believe that 2.4 H/W is a safe limit to indicate surgical treatment. When we analyzed the confidence interval of the −30% group the upper limit (2.7) was near to the lower limit of the surgical group (2.9). We believe that 2.4 is an index that form a shield from over-indication or sub-indication to repair posterior wall fracture dislocation of the acetabulum, and concerning to the group called indeterminate [5] this 2.4 index contemplate it. If we accepted an index of 2.5 we would be going outside of the confidence interval for conservative treatment and increasing the risk to not operate in cases that really required surgery. Furthermore, an index of 2.5 would fall within the interval corresponding to removal of 30% of the posterior wall. There was significant difference between removing 25% and 30% of the posterior wall and, therefore, this index cannot be 2.5. The index also cannot be lower than 2.4 because we would institute surgical treatment for cases that should be conservatively treated. Nevertheless, these findings are based on small conservative group (eight hips) and all cases (twenty two hips) presented fracture dislocation and the conservative treatment was previously determined by the senior surgeon; unfortunately, these findings may be a bias in our study.

The concept that loss of one third of the posterior wall may not be an instability factor but might give rise to future joint overload [17] is an important issue that reinforces the choice of an index of 2.4. Our findings, which presented significant difference between −25% and −30% groups are also important. On the other hand, bad results not only depend on the joint overload, but depend on the many others circumstance such as: fracture types, vascular femoral head injury, acetabular wall impact, femoral head impact, residual instability, etc. [16,21,22].

We believe that clinical criteria under anesthesia (flexion of 90 and slight adduction after performing reduction) are the gold standard [9]; however, in some cases our H/W index may be an important tool to help the surgeon to indicate or not indicate surgery. Another advantage of this method is that it is possible to make the H/W index direct from the CT scan, because the relationship between femoral head and acetabular posterior wall measurement is proportional and this ratio may be done in millimeters or centimeters.

Our study presents some points that should be addressed: our main issue is a possibly remaining instability of the hip joint, however, patients with femoral head fracture, intra-articular fragments and marginal impactions were also included. In these patients the indication for operation is given even in case of a stable joint. The two patients operated were indicated to surgery prior to the method because the size of the fragments was considered large by the surgeon. They were submitted to the index and proved to be within the proposed limits. We did not perform a prospective study and we did not perform correlation between the tomographic index calculated and hip instability. Further studies are worth to investigate this issue. Furthermore, it is important to make a clinical evaluation of the patients and consider overall the pathologic changes that occur with the fractures in order to indicate or not indicate surgery.

Our study presents a tool which is able to assist the analysis of images, especially with clinical examination, in order to provide an indication of operation, it should not be considered as a single factor to be evaluated. The advantage of this index is that it is only performed on one hip, the affected hip, unlike other different methods [9-15]. We suggest future studies to apply the new index in prospective studied. As a first study its validation could be evaluated among others patients. The validated index could also be applied in prospective and transversal studies.

## Conclusion

We indicated a tomographic acetabular index of 2.4 for the relationship of the head with the fractured posterior wall. This index is useful to assess the presence of unstable posterior wall fracture of the acetabulum.

## Competing interests

The authors declare that they have no competing interest.

## Authors’ contributions

All authors participated in the acquisition of data and revision of the manuscript. ENF, ENY, EM, TC, RYI, LMRR, CBM and CM conceived of the study, determined the design, performed the statistical analysis, interpreted the data and drafted the manuscript. VEV and LCA determined the design and drafted the manuscript. All authors read and gave final approval for the version submitted for publication.
